# Predicting incidence of hepatitis E for thirteen cities in Jiangsu Province, China

**DOI:** 10.3389/fpubh.2022.942543

**Published:** 2022-10-03

**Authors:** Tianxing Wu, Minghao Wang, Xiaoqing Cheng, Wendong Liu, Shutong Zhu, Xuefeng Zhang

**Affiliations:** ^1^School of Computer Science and Engineering, Southeast University, Nanjing, China; ^2^Jiangsu Provincial Centre for Disease Control and Prevention, Jiangsu Institution of Public Health, Nanjing, China; ^3^Chinese Field Epidemiology Training Program, Chinese Center for Disease Control and Prevention, Beijing, China

**Keywords:** hepatitis E, BiLSTM, attention, machine learning, meteorological factors, Baidu index

## Abstract

Hepatitis E has placed a heavy burden on China, especially in Jiangsu Province, so accurately predicting the incidence of hepatitis E benefits to alleviate the medical burden. In this paper, we propose a new attentive bidirectional long short-term memory network (denoted as BiLSTM–Attention) to predict the incidence of hepatitis E for all 13 cities in Jiangsu Province, China. Besides, we also explore the performance of adding meteorological factors and the Baidu (the most widely used Chinese search engine) index as additional training data for the prediction of our BiLSTM–Attention model. SARIMAX, GBDT, LSTM, BiLSTM, and BiLSTM–Attention models are tested in this study, based on the monthly incidence rates of hepatitis E, meteorological factors, and the Baidu index collected from 2011 to 2019 for the 13 cities in Jiangsu province, China. From January 2011 to December 2019, a total of 29,339 cases of hepatitis E were detected in all cities in Jiangsu Province, and the average monthly incidence rate for each city is 0.359 per 100,000 persons. Root mean square error (RMSE) and mean absolute error (MAE) are used for model selection and performance evaluation. The BiLSTM–Attention model considering meteorological factors and the Baidu index has the best performance for hepatitis E prediction in all cities, and it gets at least 10% improvement in RMSE and MAE for all 13 cities in Jiangsu province, which means the model has significantly improved the learning ability, generalizability, and prediction accuracy when comparing with others.

## 1. Introduction

Hepatitis E virus (HEV) is a quasi-enveloped virus with a single-stranded positive RNA genome belonging to the hepatitis virus family and the causative agent of hepatitis E. It is a class B notifiable infectious disease with strong contagiousness, complex transmission routes, and serious harm (1). It is also a newly emerging disease affecting humans worldwide. The clinical course of hepatitis E is often asymptomatic. Clinical symptoms include fever, anorexia, and jaundice. Extrahepatic manifestations of severe sequelae, including chronic diseases leading to liver failure and death, maybe especially in immunocompromised patients and in the presence of comorbidities (2). It was reported that pregnant women infected with HEV generally suffered from severe diseases (e.g., liver failure), and the fatality rate of pregnant women was up to 40% (3).

Hepatitis E mainly occurs in sporadic cases in developed countries and the form of epidemics in developing countries (4–6). Its epidemic characteristics are similar to hepatitis A, and it is mainly transmitted through the fecal-oral route with obvious seasonality. Hepatitis E virus is usually transmitted through drinking water and food contaminated by HEV-infected persons. Water-based epidemics are the most common, and a few are food-based outbreaks or spread through daily contact (7, 8). At the same time, the relevant literature shows that the incidence of hepatitis E is associated with meteorological factors (9).

Outbreaks of hepatitis E spread all over the world. Currently, epidemics have reported that the detection rate of HEV-IgG antibodies in some continents is as high as 50% (6), including Asia, Africa, America, and Europe, especially in Asia and Africa, where hepatitis E is endemic. China is one of the areas with a high incidence of hepatitis E, which is also one of the main diseases of adult acute viral hepatitis in China. From 2012 to 2020, the reported incidence of hepatitis E has exceeded that of hepatitis A for nine consecutive years. The meta-analysis results on the prevalence of hepatitis E in mainland China showed that the positive rates of HEV-IgG serum antibody were 27.3% in the population and 47.4% in the occupational population (10), respectively. The incidence rate of hepatitis E in Jiangsu province is also at the forefront of the country. A health economics study on hepatitis E in Jiangsu province demonstrated that the total economic burden of hepatitis E patients accounted for 60.77% of per capita disposable income (11). Its prevention and control have become an important public health issue. However, the existing information management system of disease monitoring lacks effective prediction and early warning mechanisms, which restricts the development of prevention and control of hepatitis E. Predicting the development law of the hepatitis E epidemic based on statistical analysis and mathematical models is an indispensable scientific basis for the control, prevention, and health decision-making of the hepatitis E epidemic.

Currently, the autoregressive integrated moving average (ARIMA) model is the most widely used model to predict the incidence of hepatitis E (12). Autoregressive integrated moving average can difference the data to obtain a stationary series, which makes it suitable even for some non-stationary data, but the result may be unsatisfactory because differencing the data too much can cause a large loss of information. In order to solve this problem, machine learning models, including support vector machine (SVM) (13) and gradient boosting decision tree (GBDT) (14), are introduced to predict the incidence and early warning of hepatitis E. However, the above statistical methods often fail to utilize other potential features like meteorological factors besides the historical incidence. In addition, machine learning solutions usually require complex feature engineering. Therefore, it is necessary to find the key features before training, which requires many scholars' practical experience and domain-specific knowledge to implement manually, and it has become the bottleneck of machine learning in data analysis. Deep learning begins to receive more attention due to its good performance in time series analysis and gradually surpasses and replaces traditional machine learning in time series forecasting, because it can conduct deep-processing on features automatically. Some works use a back-propagation neural network (15) or long short-term memory (LSTM) (13) to predict the incidence of hepatitis E. At present, the state-of-the-art model is LSTM used by Guo et al. (13). This model can accurately capture the features of sequential data and effectively avoid the problems of vanishing gradient and exploding gradient on traditional recurrent neural networks, so it is widely used on various prediction tasks such as wind power prediction (16) and COVID-19 prediction (17). However, it only uses the past monthly incidence rates of hepatitis E to predict the incidence rate for the next month, and it cannot correct the current prediction with the input of the next time point. Besides, meteorological factors are not considered in their model. With the rapid development of the internet, its role in the monitoring and early warning of infectious diseases has become increasingly evident in recent years. As of 2021, the number of internet users in China has reached 989 million, and the user penetration rate of the Baidu search engine is 90.9%, making it the world's largest Chinese search engine. Many studies have shown that the frequency of queries for diseases and related symptom keywords are highly correlated with the incidence, reflecting the true searchers' need trends (18, 19), so the Baidu index for hepatitis E could be utilized in predicting the incidence of hepatitis E. Thus, the LSTM model has much room for improvement.

To address the above problems, we propose a new attentive bidirectional long short-term memory (i.e., BiLSTM–Attention) model considering various meteorological factors and the Baidu index in this paper to predict the incidence of hepatitis E for all cities in Jiangsu Province. Besides, we compare our proposed model with existing models using seasonal-ARIMA with exogenous variables (SARIMAX), LSTM, BiLSTM, and GBDT, aiming to provide a scientific basis for predicting the incidence of hepatitis E more effectively, which also facilitates the development of the early warning system and the prevention strategies for hepatitis E in Jiangsu Province.

## 2. Methods

### 2.1. Data sources

In our dataset, for each city in Jiangsu province, we have monthly incidence rates of hepatitis E, monthly average temperatures (°C), monthly average water vapor pressures (100 Pa), monthly precipitations (mm), and monthly Baidu indexes. Monthly cases of hepatitis E from the 13 cities in Jiangsu Province from January 2011 to December 2019 are provided by the National Notifiable Infectious Disease Surveillance System^1^. The monthly incidence rate means the number of people who suffer from hepatitis E each month in each city in Jiangsu province per 100,000 persons. Demographic data are extracted from the statistical yearbook published by the Jiangsu provincial government. It is used to calculate monthly incidence rates. We obtain monthly average temperatures, monthly average water vapor pressures, monthly precipitations (from January 2011 to December 2019) for each city from the China Meteorological Data Service Centre. We extract the daily Baidu index of each city from the Baidu official website^2^. The monthly Baidu index is calculated by the sum of the daily Baidu index. These data are previously examined to avoid the error and exception values.

### 2.2. Experiment description

We employ a BiLSTM–Attention model to predict the incidence of hepatitis E in 13 cities in Jiangsu province, China. The bidirectional long short-term memory (BiLSTM) avoids the vanishing gradient and exploding gradient problems by the regretting mechanism. It is composed of two LSTMs with different directions, so it can preserve the information from both past and future, and the attention mechanism calculates the weight of each feature automatically, which emphasizes the effects of key features in prediction. We evaluate the existing models and our model with the dataset mentioned in Section 2.1, and explore whether meteorological factors and the Baidu index can improve our model performance. We use root mean square error (RMSE) and mean absolute error (MAE) to evaluate the prediction results, compare the performance of different models, and evaluate the influences of adding meteorological factors and the Baidu index as the additional training data. Root mean square error represents the sample standard deviation of the difference between the predicted value and the observed value. When the predicted value is completely consistent with the true value, then the RMSE is equal to 0. The greater the error, the greater the value. Given the number of months *n*, the true incidence of the *i*-th month *y*_*i*_ and the observed incidence of the *i*-th month ŷ_*i*_, the RMSE is computed as follows:


(1) $$\begin{array}{l} RMSE=\sqrt{\frac{1}{n}\overset{n}{\underset{i=1}{\sum }}{\left(y_{i}-{ŷ}_{i}\right)}^{2}} \end{array}$$

Mean absolute error is the average error between the true and predicted value. The MAE is computed as follows:


(2) $$\begin{array}{l} MAE=\frac{1}{n}\overset{n}{\underset{i=1}{\sum }}|y_{i}-{ŷ}_{i}| \end{array}$$

### 2.3. Model construction

**Figure 1:**
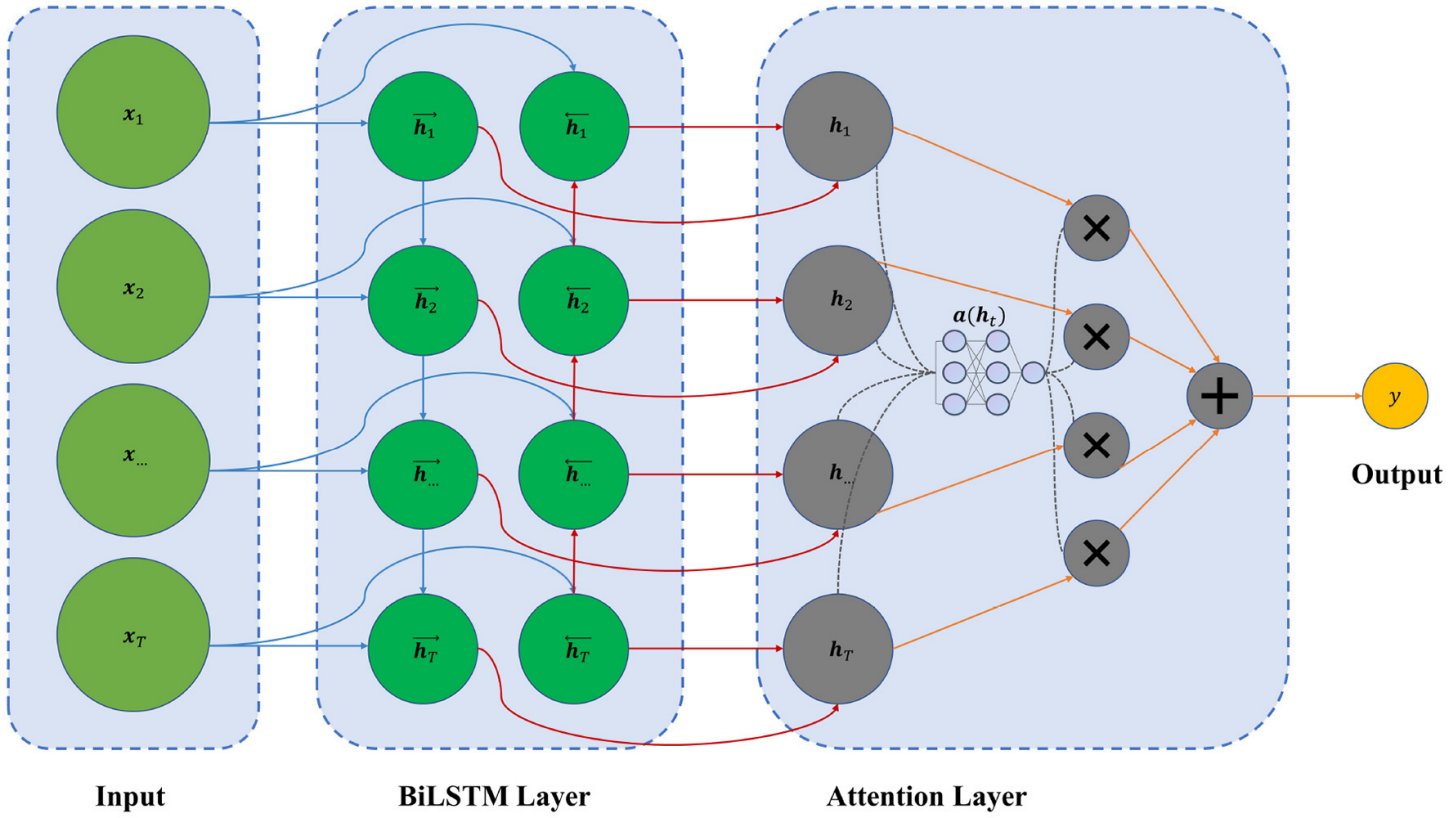
The framework of our BiLSTM–Attention model.

Figure 1 shows the overall framework of the model.

The input is the feature vectors ***x***_1_, ***x***_2_, ...,***x***_*T*_, and each vector ***x***_*t*_ can be composed of the monthly incidence rates of hepatitis E, meteorological factors (including temperature, water vapor pressure, and precipitation), and the Baidu index. After passing through a Min–Max scaler to normalize each feature vector, we use a combination of *T* months' data to make a prediction.

**Figure 2:**
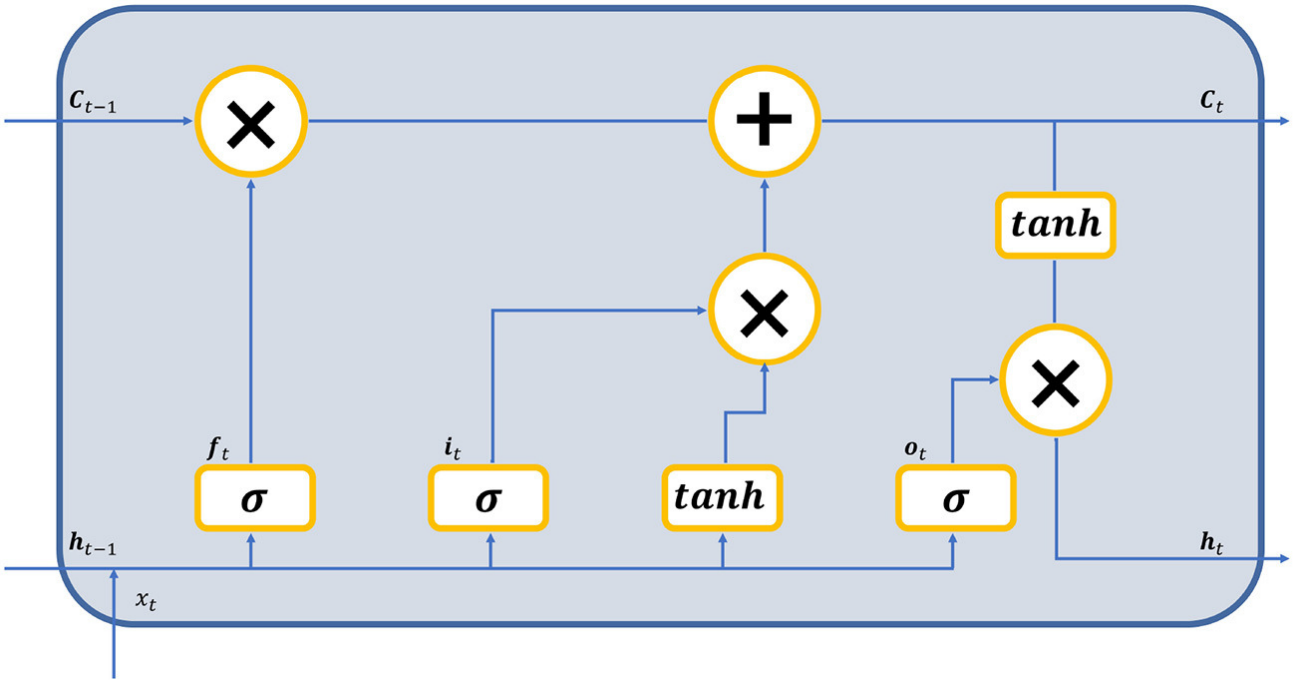
The structure of the LSTM cell.

The BiLSTM layer contains forward and backward LSTM layers, which output vectors ${\vec{h}}_{t}$ and ${\overset{⃖}{h}}_{t}$. The structure of the LSTM cell is shown in Figure 2.

During the forward step, the input vectors are fed to the LSTM cells, each of which consists of three gates. The input gate ***i***_*t*_, the forget gate ***f***_*t*_, and the output gate ***o***_*t*_ generate a value between 0 and 1 to determine the proportion of the information passing through these gates. With the current input $x_{t}\in {\mathbb{R}}^{N\times 1}$ and previous state ${\vec{h}}_{t-1}$, we get the new state ***C***_*t*_, where *N* is the size of features. Finally we get the output vector ${\vec{h}}_{t}$ of the cell. The following five equations describe the inherent logic of a forward LSTM cell:


(3) $$\begin{array}{l} i_{t}=\sigma \left(\left(W_{i}\cdot \left({\vec{h}}_{t-1}∥x_{t}\right)\right)+b_{i}\right) \end{array}$$

(4) $$\begin{array}{l} f_{t}=\sigma \left(\left(W_{f}\cdot \left({\vec{h}}_{t-1}∥x_{t}\right)\right)+b_{f}\right) \end{array}$$

(5) $$\begin{array}{l} o_{t}=\sigma \left(\left(W_{o}\cdot \left({\vec{h}}_{t-1}∥x_{t}\right)\right)+b_{o}\right) \end{array}$$

(6) $$\begin{array}{l} C_{t}=f_{t}*C_{t-1}+i_{t}*\tanh\left(\left(W_{C}\cdot \left({\vec{h}}_{t-1}∥x_{t}\right)\right)+b_{C}\right) \end{array}$$

(7) $$\begin{array}{l} {\vec{h}}_{t}=o_{t}*\tanh\left(C_{t}\right) \end{array}$$

where $W_{i},W_{f},W_{C},W_{o}\in {\mathbb{R}}^{m\times 2N}$ represent the weight matrices, *m* is the hidden size of the LSTM layer, $b_{i},b_{f},b_{C},b_{o}\in {\mathbb{R}}^{u}$ represent the bias vectors, ∥ means vector concatenation, * stands for the scalar product, σ is the sigmoid function, and tanh is the hyperbolic tangent function. Note that the backward equation can be derived similarly by replacing ${\vec{h}}_{t}$ with ${\overset{⃖}{h}}_{t}$. The following equation calculates the result of the BiLSTM layer:


(8) $$\begin{array}{l} h_{t}=c_{1}{\vec{h}}_{t}+c_{2}{\overset{⃖}{h}}_{t} \end{array}$$

where ***h***_*t*_ represents the output of the BiLSTM layer, *c*_1_ and *c*_2_ are weights of the two vectors, respectively.

We use the feed-forward attention in the attention layer. The attention mechanism gives each feature different weights, making important features with greater weights. The calculation of the attention score can be described as the following equation:


(9) $$\begin{array}{l} a\left(h_{t}\right)=tanh\left(w^{T}h_{t}+b\right) \end{array}$$

where ***a***(***h***_*t*_) is the attention score, ***w***^*T*^ and ***b*** represent the weight vector and the bias vector in the attention layer, respectively. As for the output layer, we have


(10) $$\begin{array}{l} y=\sigma \left(WH+b\right) \end{array}$$

where *y* is the output value, ***W*** is the weight vector of the output layer, ***H*** is the weighted sum of ***h***_1_, ..., ***h***_*T*_, *b* is the bias value, and σ represents the activation function, which the sigmoid function is used in this layer.

### 2.4. Model performance

We propose a new BiLSTM–Attention model to predict the incidence of hepatitis E in 13 cities in Jiangsu province, China, and evaluate the existing models and our model with the dataset mentioned in Section 2.1 (details are also given in Section 3.1), and explore whether meteorological factors and the Baidu index can improve our model performance. The result shows that our model significantly outperforms other existing models in RMSE by 33% on average and in MAE by 19.1% on average for all 13 cities, and both meteorological factors and the Baidu index can improve the prediction performance of our model by 18.7% on average, counting by RMSE, and 9.3% on average, counting by MAE, for all 13 cities. The detailed discussion can be seen in Section 3.2.

## 3. Results

### 3.1. General description

A total of 29,339 cases of hepatitis E were detected in Jiangsu Province from January 2011 to December 2019, collected by Jiangsu Provincial Center for Disease Control and Prevention. It should be noted that the hepatitis E virus belongs to the family hepeviridae, which can be classified into two subfamilies, five genera, and ten species, according the ICTV^3^, but the information of such cases of hepatitis E does not record the detailed virus types. Thus, we cannot divide the cases of hepatitis E based on virus types and conduct more fine-grained analysis. However, on the basis of Tian et al. (20), the virus sequences of the most cases of hepatitis E in Jiangsu Province are genotype 4.

**Figure 3:**
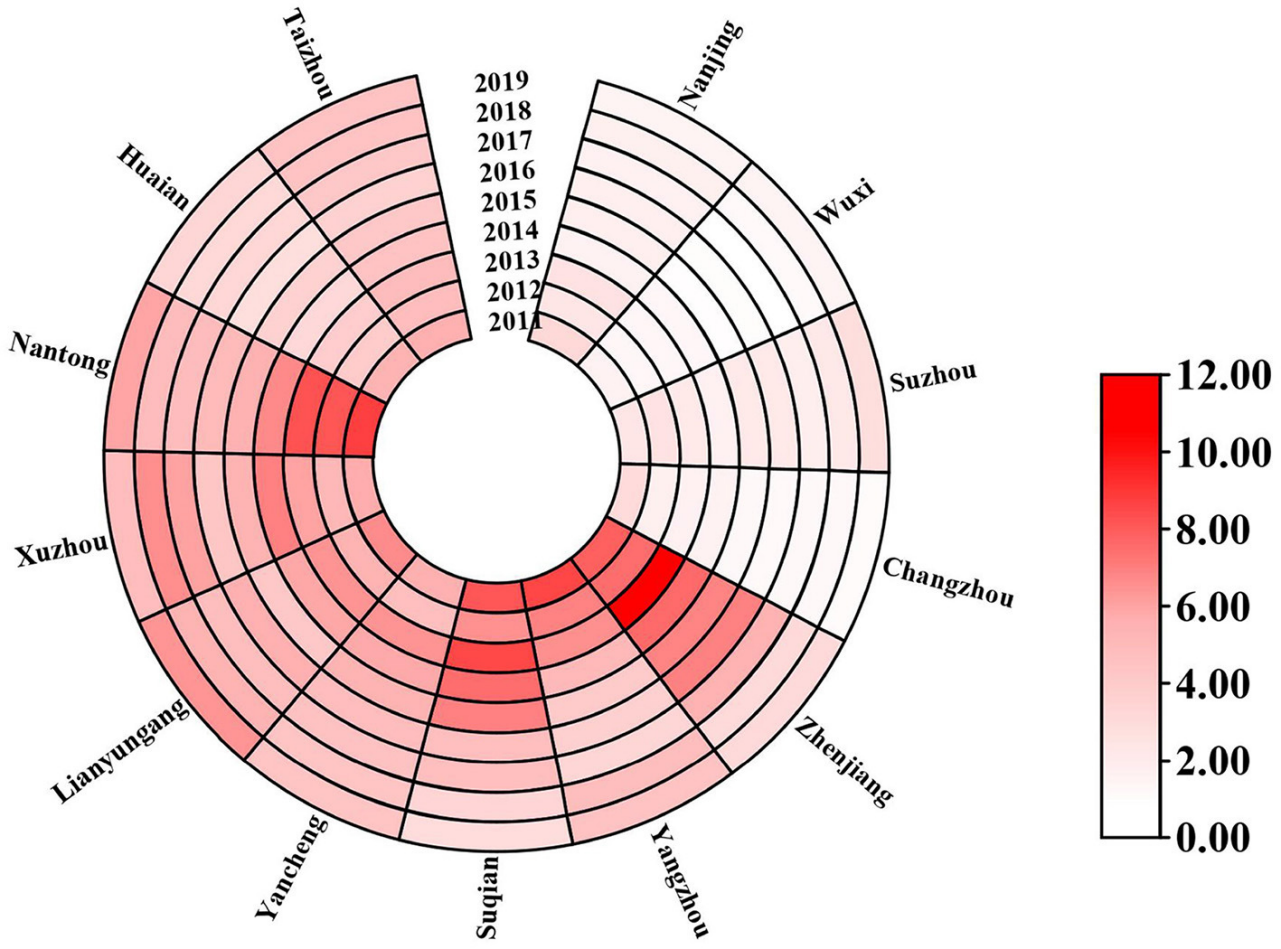
The annual incidence rates (the number of people suffering from hepatitis E each year per 100,000 persons) of hepatitis E of the 13 cities in Jiangsu Province from 2011 to 2019.

The annual incidence rates of hepatitis E (i.e., the number of people suffering from hepatitis E each year per 100,000 persons) for all 13 cities in Jiangsu Province are shown in Figure 3. We can find that Zhenjiang (6.56 per 100,000 persons), Nantong (6.48 per 100,000 persons), and Suqian (5.94 per 100,000 persons) have the highest average annual incidence rates of hepatitis E in Jiangsu Province. Besides, Wuxi (1.25 per 100,000 persons), Changzhou (1.58 per 100,000 persons), and Nanjing (2.06 per 100,000 persons) are of the lowest average annual incidence rates. The annual incidence rates of hepatitis E in Changzhou are decreasing every year, and the annual incidence rates in other cities are in a concussive decline. The monthly incidence rates of hepatitis E (i.e., the number of people suffering from hepatitis E each month per 100,000 persons) for each city varies seasonally, and the details are given in Table 1. Here, the monthly incidence rates of hepatitis E from January 2011 to June 2018 are used as the training data to train our BiLSTM–Attention model and other baselines for each city. The monthly incidence rates of hepatitis E from July 2018 to December 2019 are used as the test data to evaluate the prediction accuracy of different models.

**Table 1:** Statistics of the monthly incidence rates of hepatitis E for the 13 cities in Jiangsu province from January 2011 to December 2019.

**City**	**The average value**	**The highest value**	**The lowest value**
Nanjing	0.163	0.595	0.025
Lianyungang	0.441	1.32	0.067
Changzhou	0.123	0.588	0
Zhenjiang	0.506	1.237	0.031
Wuxi	0.101	0.267	0
Suzhou	0.181	0.483	0.047
Huai'an	0.286	1.021	0.041
Yangzhou	0.422	1.637	0.132
Xuzhou	0.442	0.982	0.079
Yancheng	0.416	0.815	0.152
Suqian	0.455	1.463	0.041
Taizhou	0.367	0.840	0.108
Nantong	0.513	1.304	0.192

Regarding meteorological factors (including monthly average temperatures, monthly average water vapor pressures, and monthly precipitations) and the Baidu index, the detail is given in Section 2.1.

All the data we collected are before 2020, and this is because the total number of people who suffer hepatitis E after 2020 is much lower than that before 2020, which is caused by the the pandemic of COVID-19. The spread of COVID-19 in mainland China takes up most of the hospitals and doctors, so the data collected from 2020 till now are far away from the true incidence rates of hepatitis E. If we use the model trained on the data collected before 2020 to predict the incidence rates of hepatitis E after 2020, it may lead to a rising bias and large error. Thus, if we try to train a model which has a good prediction performance after 2020, we need to re-train our model only on the data collected after 2020, but the data of less than three years since 2020 are insufficient to train a good deep learning model. This is why we did not predict the incidence rates of hepatitis E after 2020.

### 3.2. Model comparison

We compare our BiLSTM–Attention model with SARIMAX (21), GBDT (14), LSTM (13), and BiLSTM (22). Autoregressive integrated moving average is the traditional and the most widely used model for predicting disease incidence, but since it cannot deal with the features other than incidence and the seasonality of the hepatitis E data, we use the SARIMAX (21) (i.e., seasonal-ARIMAX), an ARIMA-based variant supporting time series prediction with seasonal components as an important baseline. The decision tree based GBDT model and recurrent neural network based LSTM model have already been utilized in predicting the incidence of hepatitis E, so they are also taken as the baselines. The BiLSTM model is a variant of our BiLSTM–Attention model without using the attention mechanism. For SARIMAX (21), GBDT (14), and LSTM (13), we set the hyper parameters according to previous studies. The detailed parameter setting for SARIMAX, GBDT, LSTM, BiLSTM, and BiLSTM–Attention model is provided in Table 2.

**Table 2:** The parameter setting for different models.

**Model**	**Parameter**	**Range**
SARIMAX	Number of time lags, order of MA model (*p, q*)	{(1, 1), (1, 2), ..., (3, 3)}
	AR, MA terms (seasonal part) (*P, Q*)	{(0, 0), (0, 1), ..., (3, 3)}
	Degree of differencing *d*	{0, 1}
	Differencing term (seasonal part) *D*	{1}
	Number of periods *m*	{12}
GBDT	*n* Estimators	{100, 200, 300}
	Learning rate	{0.1, 0.2, 0.5}
	Max depth	{1, 2, 3, 4, 5}
LSTM, BiLSTM, BiLSTM–Attention	Batch size	{8, 16, 32}
	Epochs	{64, 128, 256, 512}
	LSTM hidden size	{2, 4, 8, 16, 32, 64, 128}
	Validation split	{0.1}
	Optimizer	{Adam, SGD}
	Loss	{MSE, MAE}

AR, auto regression; MA, moving average; Adam (23), an algorithm for first-order gradient-based optimization of stochastic objective functions based on adaptive estimates of lower-order moments; SGD, stochastic gradient descent; MSE, mean-squared error; MAE, mean absolute error.

**Figure 4:**
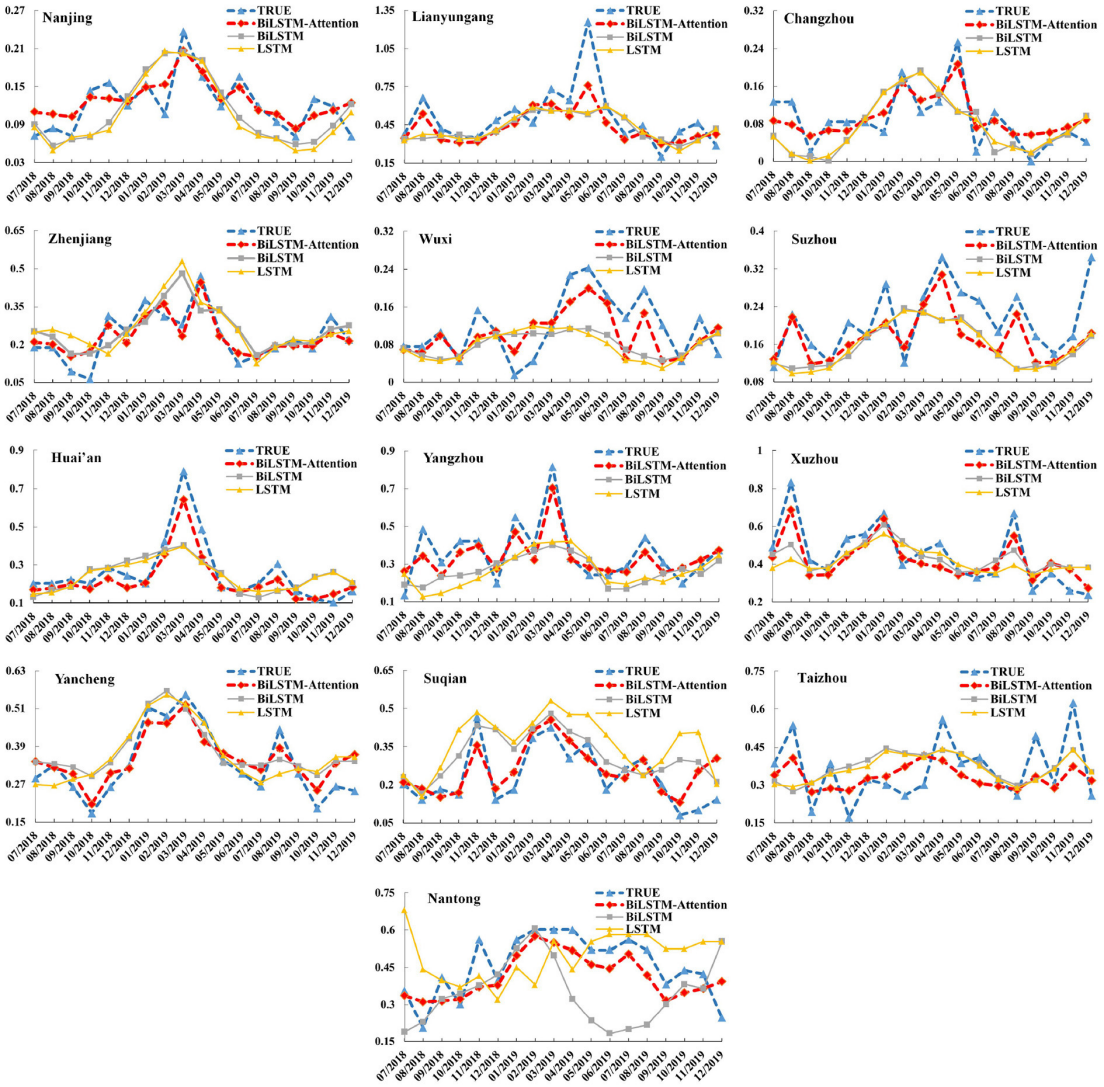
The monthly incidence rates (including the true results and predicted ones of BiLSTM–Attention, BiLSTM, and LSTM) of hepatitis E for the 13 cities in Jiangsu Province (x-axis: month, y-axis: incidence rate).

We carefully optimized the hyperparameters using beam search for each city's model. The comparison results for each city are shown in Table 3, and we can see that the BiLSTM–Attention model gets the lowest RMSE and the MAE values for all 13 cities and at least 10% RMSE and MAE improvement for each city in Jiangsu province compared with other baselines. For all the 13 cities in Jiangsu Province, Figure 4 records the true monthly incidence rates and predicted monthly incidence rates of BiLSTM–Attention, BiLSTM, and LSTM (these three models have the best RMSE and MAE according to the Table 3) from July, 2018 to December, 2019. We can find that the overall predicted results of BiLSTM–Attention are the closest to the true results, especially for some peak values.

**Table 3:** The comparison results of different models on predicting monthly incidence rates of hepatitis E for the 13 cities in Jiangsu province.

**City**	**Metric**	**Our model**	**SARIMAX**	**BiLSTM**	**LSTM**	**GBDT**
Nanjing	RMSE	0.025	0.060	0.046	0.049	0.074
	MAE	0.143	0.218	0.197	0.201	0.238
Lianyungang	RMSE	0.150	0.253	0.210	0.205	0.256
	MAE	0.335	0.396	0.368	0.363	0.398
Changzhou	RMSE	0.033	0.093	0.068	0.064	0.068
	MAE	0.170	0.290	0.231	0.227	0.240
Zhenjiang	RMSE	0.044	0.287	0.088	0.102	0.261
	MAE	0.188	0.513	0.267	0.285	0.467
Wuxi	RMSE	0.047	0.089	0.072	0.078	0.081
	MAE	0.192	0.272	0.242	0.251	0.248
Suzhou	RMSE	0.060	0.086	0.085	0.085	0.069
	MAE	0.212	0.266	0.263	0.264	0.239
Huai'an	RMSE	0.063	0.174	0.128	0.126	0.160
	MAE	0.214	0.386	0.306	0.299	0.344
Yangzhou	RMSE	0.073	0.208	0.166	0.176	0.155
	MAE	0.249	0.422	0.361	0.371	0.339
Xuzhou	RMSE	0.075	0.134	0.116	0.137	0.145
	MAE	0.254	0.336	0.296	0.307	0.333
Yancheng	RMSE	0.050	0.157	0.069	0.073	0.112
	MAE	0.208	0.354	0.245	0.239	0.303
Suqian	RMSE	0.071	0.318	0.119	0.168	0.246
	MAE	0.236	0.535	0.303	0.367	0.440
Taizhou	RMSE	0.107	0.151	0.129	0.123	0.189
	MAE	0.296	0.370	0.328	0.322	0.386
Nantong	RMSE	0.086	0.171	0.196	0.155	0.122
	MAE	0.272	0.364	0.391	0.354	0.315

### 3.3. Influence of meteorological factors and Baidu index

**Figure 5:**
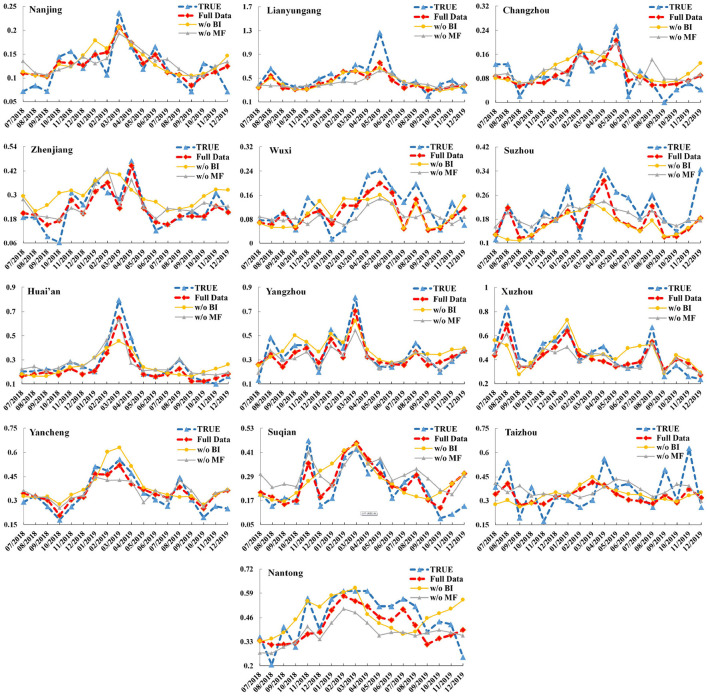
The monthly incidence rates (including the true results and predicted ones of the BiLSTM–Attention model with full training data, w/o BI, and w/o MF) of hepatitis E for the 13 cities in Jiangsu Province (x-axis: month, y-axis: incidence rate).

To test the influence of adding meteorological factors and the Baidu index in the feature vectors (i.e., the part of training data), we only used (1) the incidence data and meteorological factors (i.e., without Baidu index, denoted as w/o BI), and (2) the incidence data and Baidu index (i.e., without meteorological factors, denoted as w/o MF), as the training data, respectively. The comparison results are shown in Table 4, and we can see that if we use the full data including the incidence data, meteorological factors, and Baidu index for training, the RMSE and MAE of our BiLSTM–Attention model will be greatly improved compared with using w/o BI or w/o MF for training. This also reflects the effectiveness of meteorological factors and the Baidu index. Figure 5 records the true monthly incidence rates and predicted monthly incidence rates of our BiLSTM–Attention model with full training data, w/o BI, and w/o MF from July, 2018 to December, 2019. We can find that the overall predicted results of the BiLSTM–Attention model with full training data (denoted as full data) are the closest to the true results, especially for peak values.

**Table 4:** The comparison results of our BiLSTM-Attention model with different training data on predicting monthly incidence rates of hepatitis E for the 13 cities in Jiangsu province.

**City**	**Metric**	**Full data**	**w/o BI**	**w/o MF**
Nanjing	RMSE	0.025	0.033	0.034
	MAE	0.143	0.167	0.175
Lianyungang	RMSE	0.150	0.171	0.200
	MAE	0.335	0.344	0.362
Changzhou	RMSE	0.033	0.057	0.047
	MAE	0.170	0.218	0.204
Zhenjiang	RMSE	0.044	0.104	0.068
	MAE	0.188	0.288	0.241
Wuxi	RMSE	0.047	0.062	0.058
	MAE	0.192	0.233	0.218
Suzhou	RMSE	0.060	0.078	0.061
	MAE	0.210	0.252	0.212
Huai'an	RMSE	0.063	0.105	0.077
	MAE	0.214	0.269	0.228
Yangzhou	RMSE	0.073	0.096	0.087
	MAE	0.240	0.281	0.249
Xuzhou	RMSE	0.075	0.118	0.084
	MAE	0.250	0.306	0.254
Yancheng	RMSE	0.05	0.074	0.067
	MAE	0.208	0.258	0.243
Suqian	RMSE	0.071	0.106	0.086
	MAE	0.236	0.293	0.273
Taizhou	RMSE	0.107	0.133	0.116
	MAE	0.296	0.328	0.303
Nantong	RMSE	0.086	0.120	0.115
	MAE	0.272	0.306	0.320

## 4. Discussion

Hepatitis E is considered a major public health problem in China, seriously threatening people's health. From January 2011 to December 2019, the monthly incidence rate in Jiangsu Province fluctuated between 0.12 and 0.83 per 100,000 persons, with significant seasonal variation.

The incidence of hepatitis E in 13 cities in Jiangsu Province is mainly sporadic, and the disease occurs every month of the year. The monthly incidence rate of hepatitis E from 2011 to 2019 showed a slow upward trend, decreasing slowly before 2016 and rising slowly after 2017. In 2012, the world's first hepatitis E vaccine (HEV 239, Xiamen Innovax Biotech, Xiamen) was launched in China (24). However, vaccination rates remain relatively low in Jiangsu Province, and the risk of infection in the population remains high. At the same time, our findings suggest that the incidence of hepatitis E varies by region. According to the Jiangsu Statistical Yearbook, Zhenjiang and Yangzhou have lower GDP rankings. In contrast, Suzhou, Wuxi, and Changzhou have higher GDP rankings, which may be an important reason for the uneven distribution of disease burden in Jiangsu Province. In addition, different climatic environments, population size, urban environment construction, and living habits may be another reason for the uneven distribution of disease burden in Jiangsu Province. Therefore, it is particularly important to consider multiple perspectives and dimensions to do epidemic early warning and strengthen epidemic monitoring.

This paper attempts to use the monthly incidence rates of hepatitis E from January 2011 to December 2019 to establish different prediction models, including SARIMAX, GBDT, LSTM, BiLSTM, and the BiLSTM–Attention model with meteorological factors and the Baidu index. The experimental results show that our BiLSTM–Attention model has achieved the best performance (evaluated by RMSE and MAE) for the 13 cities in Jiangsu province.

Autoregressive integrated moving average is one of the most popular and convenient linear models for time series forecasting, which comprehensively considers the periodicity, seasonality, randomness, and other factors of infectious diseases (25–27). Recently, some researchers used the ARIMA model to predict the incidence of hepatitis E in Jiangsu and Shanghai, consistent with the results in this paper, the model did not exhibit satisfactory performance, it may be because the linear equation cannot fully and effectively extract the epidemic trend information, resulting in unsatisfactory model fitting and prediction effects. In order to solve the above problems, some researchers used machine learning models to predict the incidence of hepatitis E. Ren et al. (15) employed a combined model using an ARIMA model and a back-propagation artificial neural network to forecast the incidence of hepatitis E in Shanghai, Guo et al. (13) adopted the ARIMA model, SVM, and LSTM model to forecast the incidence of hepatitis E in Shandong province, the results demonstrated that non-linear models outperform linear models (ARIMA). In this study, we also found that GBDT, LSTM, BiLSTM, and the BiLSTM–Attention are better than the ARIMA model. Some researchers have indicated that infectious diseases are sensitive to climate, which may influence the survival, spread, and host susceptibility of infectious pathogens in the environment. In recent years, the influence of relative humidity, temperature, rainfall, and other meteorological factors on the prevalence of hepatitis E has received extensive attention. Peng et al. (14) developed GBDT and Random Forest to forecast the incidence of hepatitis E, and the prediction performance of the model with water quality and meteorological data has been significantly improved. In our study, in terms of meteorological factors, our results suggest that the incidence of hepatitis E in Jiangsu Province is related to monthly average temperatures, monthly average water vapor pressures, and monthly precipitations, and the BiLSTM–Attention model with meteorological factors gets 30.6% RMSE improvement and 17% MAE improvement on average for each city. When the temperature drops, the resistance of susceptible people is relatively low, and the low temperature is conducive to the spread of the virus. Around the Spring Festival in winter, the number of people going out and eating together increases, so the probability of exposure to animal food increases, resulting in a rise in the incidence. The increase in monthly precipitations may be conducive to the water-borne transmission of hepatitis E, resulting in a rise in the number of cases. The specific mechanism of the effect of water vapor pressure on the onset of hepatitis E is unclear and needs to be further studied.

With the development of the Internet and the popularity of smartphones, big data analysis is increasingly applied to the prediction and early warning of infectious diseases. The research outside China is often based on the Google search engine to predict the trend of dengue fever (28) and influenza (29). The research in China [e.g., influenza (30), dengue fever (31), hand, foot, and mouth disease (19), and etc.] often uses the Baidu search index and predicts with different mathematical models. However, there is no study on the prediction model of hepatitis E incidence in Jiangsu Province based on the Baidu search index. In this study, the results show that adding the Baidu index can improve the accuracy of model prediction, and the BiLSTM–Attention model with the Baidu index gets an average of 19.8% RMSE improvement and 10.3% MAE improvement for each city, suggesting that real-time monitoring and prediction should be more suitable for the Baidu index.

Our model performed the best in both the model fitting and prediction phases. On the one hand, the RMSE and MAE are the lowest in our model for the 13 cities; on the other hand, the predicted incidence was consistent with the actual incidence, and the simulated seasonal changes at the peak of winter and the lowest point of autumn also coincide with reality. However, the incidence of hepatitis E is influenced by many environmental and natural factors, which are dynamic and may evolve. Therefore, the parameters of the model should be periodically re-evaluated based on continuously updated data to maintain long-term sustainability and accuracy.

This study also has some limitations. Baidu keywords are affected by the cultural and educational level of netizens and the health needs of individuals, resulting in a wide range of keywords; Besides, people may also be affected by media reports, so the Baidu index has a media effect, resulting in the vacuousness of the Baidu index.

## 5. Conclusion

This paper proposed a new BiLSTM–attention model with various meteorological factors and the Baidu index to predict the monthly incidence rates of hepatitis E in the 13 cities in Jiangsu Province, China. Compared with the baseline models, including SARIMAX, GBDT, LSTM, and BiLSTM, our BiLSTM–attention model has the lowest RMSE and MAE. Experiments also show that adding meteorological factors including temperature, water vapor pressure, and precipitation or Baidu index into the training data can significantly improve the prediction results for all 13 cities.

As for the future work, we will try to apply our prediction model in real-time scenarios. Besides, we plan to find more external factors which influence the prediction of hepatitis E using machine learning models, and the correlations between such factors and the spread of hepatitis E.

## Data availability statement

The raw data supporting the conclusions of this article will be made available by the authors, without undue reservation.

## Author contributions

TW and MW conceived and designed the study, performed the analysis, and wrote the manuscript. XC, WL, SZ, and XZ contributed to the revision of the manuscript draft. All authors read and approved the final manuscript.

## Funding

This work is supported by the NSFC (Grant No. 62006040), the Project for the Doctor of Entrepreneurship and Innovation in Jiangsu Province (Grant No. JSSCBS20210126), the Fundamental Research Funds for the Central Universities, and ZhiShan Young Scholar Program of Southeast University.

## Conflict of interest

The authors declare that the research was conducted in the absence of any commercial or financial relationships that could be construed as a potential conflict of interest.

## Publisher's note

All claims expressed in this article are solely those of the authors and do not necessarily represent those of their affiliated organizations, or those of the publisher, the editors and the reviewers. Any product that may be evaluated in this article, or claim that may be made by its manufacturer, is not guaranteed or endorsed by the publisher.

## Footnotes

^1^ http://www.cdpc.chinacdc.cn

^2^ http://index.baidu.com

^3^ https://ictv.global/report/chapter/hepeviridae/hepeviridae
